# Preoperative intravenous rehydration for patients with pheochromocytomas and paragangliomas: is it necessary? A propensity score matching analysis

**DOI:** 10.1186/s12871-020-01212-6

**Published:** 2020-11-30

**Authors:** Hao Kong, Jiao-Nan Yang, Jie Tian, Nan Li, Yu-Xiu Zhang, Peng-Cheng Ye, Xue-Ying Li, Zheng Zhang

**Affiliations:** 1 Department of Anesthesiology and Critical Care Medicine, Peking University First Hospital, No. 8 Xishiku Street, Beijing, 100034 China; 2 Key Laboratory of Carcinogenesis and Translational Research (Ministry of Education/Beijing), Department of Anesthesiology, Peking University Cancer Hospital & Institute, Beijing, 100142 China; 3 Department of Urology, Peking University First Hospital, Beijing, 100034 China; 4 Department of Biostatistics, Peking University First Hospital, Beijing, 100034 China

**Keywords:** Pheochromocytoma, Paraganglioma, Intravenous rehydration, Hemodynamic instability, Outcome

## Abstract

**Background:**

Preoperative intravenous rehydration for patients with pheochromocytomas and paragangliomas (PPGLs) is widely used in many medical centers, but its usefulness has not been well evaluated. The objective of this study was to compare the perioperative hemodynamics and early outcome between patients who received preoperative intravenous rehydration and those without for resection of PPGLs.

**Methods:**

In this retrospective propensity score-matched cohort study, the data of patients who underwent surgery for PPGLs were collected. Patients were divided into two groups depending on whether they received or did not receive intravenous rehydration preoperatively. The primary endpoint was intraoperative hypotension, described as the cumulative time of mean arterial pressure < 65 mmHg averaged by surgery duration.

**Results:**

Among 231 enrolled patients, 113 patients received intravenous rehydration of ≥2000 ml daily for ≥2 days before surgery and 118 patients who did not have any intravenous rehydration before surgery. After propensity score matching, 85 patients remained in each group. The median cumulative time of mean arterial pressure < 65 mmHg averaged by surgery duration was not significantly different between rehydrated patients and non-rehydrated patients (median 3.0% [interquartile range 0.2–12.2] versus 3.8% [0.0–14.2], median difference 0.0, 95%CI − 1.2 to 0.8, *p* = 0.909). The total dose of catecholamines given intraoperatively, volume of intraoperative fluids, intraoperative tachycardia and hypertension, percentage of patients who suffered from postoperative hypotension, postoperative diuretics use, and postoperative early outcome between the two groups were not significantly different either.

**Conclusions:**

For patients with PPGLs, preoperative intravenous rehydration failed to optimize perioperative hemodynamics or improve early outcome.

**Supplementary Information:**

The online version contains supplementary material available at 10.1186/s12871-020-01212-6.

## Background

Pheochromocytomas and paragangliomas (PPGLs) are rare catecholamine-producing neuroendocrine tumors originating from the chromaffin cells of the adrenal medulla or extra-adrenal paraganglia. Surgery is the only curative therapy for PPGLs but can precipitate an increased risk of hemodynamic instability and major morbidities [1].

PPGLs are associated with profound sympathetic vasoconstriction, and α-blockade has been the mainstay of preoperative management [2]. However, relative intravascular hypovolemia can occur after α-blockade due to vascular dilation, resulting in postural hypotension and post-resection hypotension [3, 4]. Therefore, intravenous rehydration is very likely to be needed before or concurrent with the commencement of α-blockade to prevent severe hypotension. Retrospective data have suggested that preoperative volume expansion achieved by saline infusion or increased water intake can reduce the risk of postural hypotension and perioperative hypotension by optimizing intravascular status [4–7]. Bai and colleagues [8] developed a nomogram for preoperative prediction of intraoperative hemodynamic instability (IHD) related to surgical treatment of pheochromocytoma. They found that an absence of preoperative volume expansion was an effective predictor for IHD involvement. However, only half of their patient cohort received adequate α-blockade preoperatively.

Recently, an increasing number of scholars have questioned the necessity of preoperative fluid replacement. First, meticulous intraoperative management is likely more pivotal than preoperative preparation in achieving adequate control of IHD and safe clinical outcome [3, 9, 10]. A recent meta-analysis assessed the benefit of preoperative α-blockade before adrenalectomy for pheochromocytoma, and found no difference in mortality, cardiovascular complications, mean maximal intraoperative blood pressure, or mean maximal intraoperative heart rate between patients with α-blockade and those without [11]. Second, time-consuming preoperative preparation seems to be redundant on the premise of a mortality rate of only 0.5% and a morbidity rate of 5% after surgical treatment for PPGLs [12]. Both rates were even lower than in patients who underwent noncardiac surgery reported by previous large sample studies [13, 14]. Third, the main reason for post-resection hypotension is vasoplegia rather than insufficient blood volume [15]. Fourth, the detrimental effects of volume overload are being recognized gradually and heeded by anesthesiologists and surgeons [16].

Given a lack of solid evidence on the efficacy of preoperative intravenous rehydration, we conducted a cohort study to assess the usefulness of preoperative rehydration on perioperative hemodynamics and early outcome in patients undergoing surgical treatment for PPGLs.

## Methods

### Ethical approval of the study protocol

The study protocol was approved by the Clinical Research Ethics Committee of Peking University First Hospital (Beijing, China) on 7 August 2019 (approval number: 2019 [182]). Written informed consent from all enrolled patients was waived. However, the privacy of patients was protected strictly. Our study adhered to Enhancing the QUAlity and Transparency Of health Research (EQUATOR) guidelines.

### Patient recruitment

We retrospectively screened patients who underwent surgical treatment for PPGLs from December 2004 to December 2018 from the electronic medical records (EMRs) of our hospital. The inclusion criteria were patients: (i) aged ≥18 years; (ii) who had undergone laparotomy or laparoscopic surgery; (iii) who received α-blockade > 7 days; (iv) whose diagnosis of PPGLs was confirmed by pathology examinations. The exclusion criteria were patients: (i) who had undergone transurethral surgery; (ii) could not tolerate oral intake of fluids; (iii) complicated by congestive heart failure or/and renal insufficiency; (iv) with bilateral PPGLs; (v) incomplete perioperative data in EMRs; (vi) who were rehydrated for < 2 days and/or had < 2000 ml daily.

Patients were divided into two groups. One group did not receive any intravenous rehydration preoperatively. The other group was rehydrated with ≥2000 ml daily for ≥2 days preoperatively.

### Perioperative care

After the diagnosis of PPGLs, alpha-blockade was administered for at least 1–2 weeks before resection using blood pressure-guided dose titration with a target blood pressure of lower than 140/90 mmHg. If episodes of tachycardia occurred, β-blockade was also employed. During this period, consumption of a high-sodium diet and oral intake of fluids were encouraged. The decision to rehydrate, the number of days of fluid replacement, and the volume of fluids infused daily were determined by endocrinologists or operating surgeons based on personal experience.

In the operating theatre, a large-bore peripheral intravenous catheter and a central venous catheter were established for all patients. An intra-arterial catheter was inserted routinely to monitor beat-to-beat intraoperative hemodynamics. To reduce the risk of post-induction hypotension, 500–1000 ml of fluids were infused routinely before the induction of anesthesia. All patients were intubated under general anesthesia. Before tumor resection, patients were subjected to mild volume overload (central venous pressure > 8 cm H_2_O and/or stroke volume variation < 6%) to attenuate relative hypovolemia after vessel ligation, except for patients with cardiac or renal insufficiency. To manage undesirable hypertensive crisis, a combination of intravenous doses of phentolamine and esmolol was administered. After tumor removal, epinephrine, norepinephrine, and/or dopamine were used in cases of hypotension.

Upon the end of the surgical procedure, patients were transferred to the post-anesthesia care unit or intensive care unit (ICU) depending on their physical status and hemodynamics. In the ward, patients were monitored for ≥6 h after surgery. Blood pressure was measured every 15 min. In the ICU, blood pressure was monitored continuously by the intra-arterial catheter until discharge from the ICU. If hypotension was prolonged despite adequate fluid replacement therapy, catecholamine administration was continued.

### Data collection and outcome

Data were collected retrospectively from the EMR system of Peking University First Hospital and comprised demographic characteristics (age, sex, height, weight), preoperative data (surgical diagnosis, comorbidity, laboratory results, location and diameter of the tumor), intraoperative data (durations of anesthesia and surgery; anesthetic method; use of vasoactive drugs, fluid infusion, and blood transfusion; hemodynamic fluctuations), postoperative data (ICU admission, duration of vasopressor use, complications, duration of hospital stay (DOHS)). Hemodynamic data were obtained from the anesthesia information system, which captured and stored parameters every 10 s in real-time. For each patient, the collected hemodynamic data were stored in a separate Excel™ file (Microsoft, Redmond, WA, USA). Hemodynamic data were analyzed by Python 3.7.0 (Python Software Foundation, Beaverton, OR, USA).

The primary endpoint of our study was intraoperative hypotension, described as the cumulative time of mean arterial pressure < 65 mmHg, which was expressed as a percentage of surgery duration. The secondary endpoints were: (i) other perioperative hemodynamic parameters, including volume of intraoperative fluids, the total dose of intraoperative catecholamine (calculated as total equivalent dose = [dopamine dose] + [dobutamine dose] + [epinephrine dose × 100] + [norepinephrine dose × 100]) [17], intraoperative hypertension and tachycardia (described as the cumulative time of systolic arterial pressure > 160 mmHg and heart rate > 90 beats/min averaged by surgery duration, respectively), and percentage of patients suffering from postoperative hypotension (defined as hypotension that necessitated continuous vasopressor support to maintain systolic blood pressure [SBP] > 90 mmHg after surgery); (ii) percentage of patients received diuretics after surgery; (iii) early outcome during hospitalization, including postoperative complications and mortality, the frequency of mechanical ventilation, frequency of ICU admission, and DOHS.

### Statistical analyses

Matching of propensity scores was undertaken to control potential confounding factors and to obtain a baseline-balanced retrospective cohort. Twenty clinically relevant variables were used as covariables to construct a logistic regression model to calculate the propensity score. These variables were selected a priori and were: age; sex; body mass index; Charlson Comorbidity Index [18]; the presence of typical symptoms; tumors with elevated serum catecholamine; maximal diameter of the tumor; tumor origin; peak SBP before α-blockade; type of α-blockade (selective or non-selective); duration of α-blockade; preoperative β-blockade; other types of preoperative antihypertension therapy; year of surgery; surgical approach (open or laparoscopic); type of anesthesia (general or combined epidural–general); duration of surgery; intraoperative dose of phentolamine; intraoperative dose of esmolol; estimated blood loss.

We carried out a one-to-one matching using the nearest-neighbor method within a caliper width equal to 0.2 of the standard deviation of the logit of the propensity score. Standardized differences (SDs) calculated before and after propensity score matching (PSM) were used to assess the ‘balance’ between the two groups. An absolute SD ≥0.258 (i.e., $1.96\times \sqrt{\left(n1+n2\right)/\left(n1\times n2\right)}$) calculated by the formula published by Austin and colleagues [19] was considered to be ‘unbalanced’.

For endpoints, continuous variables with a Gaussian distribution were presented as the mean and standard deviation and were compared using the Student’s *t*-test, otherwise, they were presented as median and interquartile range (IQR) and were compared using the Mann–Whitney *U*-test. Categorical variables were presented as numbers and proportions and were analyzed by the chi-square test or Fisher’s exact test. The median difference (and 95% confidence interval (CI)) between two groups was calculated by the Hodges–Lehmann estimator. A two-sided *p* < 0.05 was considered significant.

We wished to evaluate the modifying effects of baseline variables on the association between preoperative intravenous rehydration and the primary endpoint. Hence, we used the Z-test to compare the difference between the two regression coefficients from subgroup analysis using the following equation [20]:

$\mathrm{Z}=\frac{\beta 1-\beta 2}{\sqrt{\mathrm{SE}{\left(\beta 1\right)}^2+ SE{\left(\beta 2\right)}^2}}$. A two-sided *p* < 0.10 was considered significant.

Statistical analyses were carried out with SPSS 22 (IBM, Armonk, NY, USA) and the free software package “R” version 2.15.3 including the “SPSS Statistics Essentials for R 22.0” and “psmatching 3.04” plugin.

## Results

### Patient recruitment

**Fig. 1:**
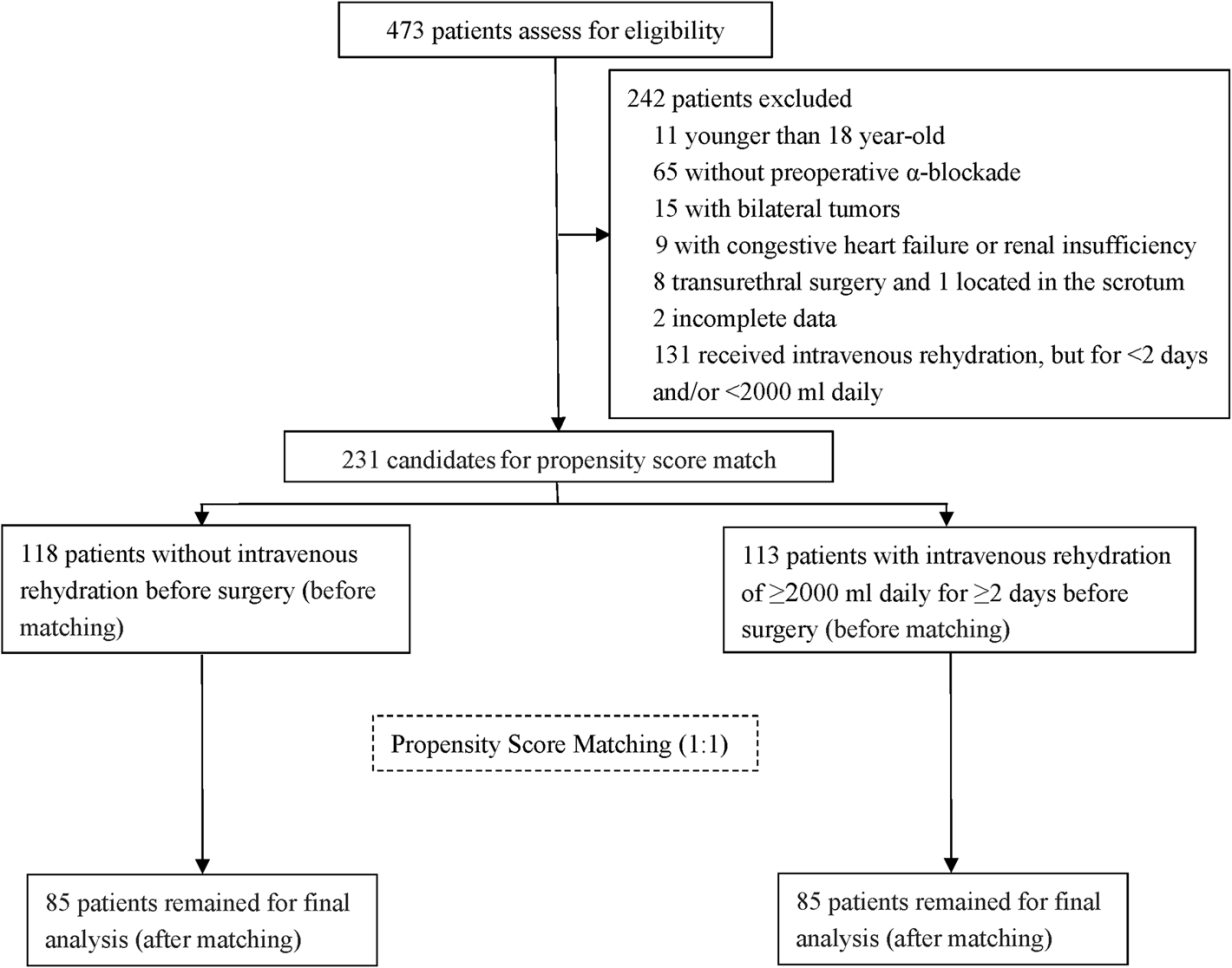
Flowchart of the study

A total of 473 patients underwent surgery for PPGLs from December 2004 to December 2018. Among them: 11 were excluded for being younger than 18 years; 65 for not undergoing preoperative α-blockade; 15 for having bilateral tumors; nine for being complicated with congestive heart failure or renal insufficiency; eight for having undergone transurethral surgery; one for having superficial surgery in the scrotum; two for having incomplete data. Besides, 131 patients who received intravenous rehydration, but for < 2 days and/or it being < 2000 ml daily, were excluded. Of the remaining 231 patients, 113 patients received intravenous rehydration of ≥2000 ml daily for ≥2 days before surgery, and 118 patients did not have any intravenous rehydration before surgery. After PSM, 85 patients remained in each group, providing a total sample of 170 patients for evaluation (Fig. 1).

### Characteristics of patients before and after PSM

Before PSM, compared with patients who did not receive intravenous rehydration, patients who received intravenous rehydration presented more typical symptoms (SD = 0.262). Besides, the year of surgery was significantly unbalanced between the two groups (SD = 0.419). After PSM, all confounding variables were well balanced (Table 1).

**Table 1:** Preoperative variables for propensity score matching

Variable	Full cohort (***n*** = 231)			Matched cohort (***n*** = 170)		
	Rehydrated group (***n*** = 113)	Non-rehydrated group (***n*** = 118)	SD ^**a**^	Rehydrated group (***n*** = 85)	Non-rehydrated group (***n*** = 85)	SD ^**a**^
**Demographics**						
Age, years	46.3 ± 14.1	46.5 ± 13.9	−0.012	45.4 ± 13.6	46.7 ± 14.5	−0.090
Male sex	48 (42.5%)	51 (43.2%)	−0.015	32 (37.6%)	37 (43.5%)	−0.118
Body mass index, kg/m^2^	23.0 ± 3.0	23.4 ± 3.2	−0.106	22.8 ± 3.2	23.1 ± 3.0	−0.081
**Charlson Comorbidity Index**	2.0 (2.0–3.0)	2.0 (2.0–2.0)	0.159	2.0 (2.0–2.0)	2.0 (2.0–2.0)	−0.038
**Features of PPGLs**						
With typical symptoms ^b^	70 (61.9%)	58 (49.2%)	**0.262**	47 (55.3%)	44 (51.8%)	0.072
With elevated serum catecholamine	80 (70.8%)	73 (61.9%)	0.196	54 (63.5%)	56 (65.9%)	−0.052
Maximal tumor diameter, cm	5.0 (4.0–6.7)	5.0 (3.9–6.9)	0.011	5.0 (4.0–6.9)	5.0 (4.0–6.8)	0.041
Origin of tumor			0.002			0.113
Adrenal gland	88 (77.9%)	92 (78.0%)		62 (72.9%)	66 (77.6%)	
Paraganglia	25 (22.1%)	26 (22.0%)		23 (27.1%)	19 (22.3%)	
Peak SBP before α-blockade, mm Hg	180 (160–210)	178 (140–200)	0.253	180 (148–200)	180 (150–205)	−0.055
**Preoperative preparation**						
Type of α-blockade			−0.074			0.000
Selective	59 (52.2%)	66 (55.9%)		49 (57.6%)	49 (57.6%)	
Non-selective	54 (47.8%)	52 (44.1%)		36 (42.4%)	36 (42.4%)	
Duration of α-blockade, days	19.0 (13.5–30.0)	17.0 (11.0–30.0)	0.021	19.0 (13.0–30.0)	20.0 (11.0–34.0)	−0.141
β-blockade	27 (23.9%)	24 (20.3%)	0.083	17 (20.0%)	18 (21.2%)	−0.027
Other antihypertensive therapy ^c^	38 (33.6%)	41 (34.7%)	−0.024	31 (36.5%)	30 (35.3%)	0.025
**Year of surgery**			**0.419**			0.026
2004–2008	16 (14.2%)	34 (28.8%)		16 (18.8%)	16 (18.8%)	
2009–2013	37 (32.7%)	34 (28.8%)		28 (32.9%)	27 (31.8%)	
2014–2018	60 (53.1%)	50 (42.4%)		41 (48.2%)	42 (49.4%)	
**Intraoperative data**						
Surgical approach			−0.021			0.049
Open	41 (36.3%)	44 (37.3%)		34 (40.0%)	32 (37.6%)	
Laparoscopic ^d^	72 (63.7%)	74 (62.7%)		51 (60.0%)	53 (62.4%)	
Type of anesthesia			0.070			−0.072
General	69 (61.1%)	68 (57.6%)		49 (57.6%)	52 (61.2%)	
Epidural + general	44 (38.9%)	50 (42.4%)		36 (42.4%)	33 (38.8%)	
Duration of surgery, min	119 (70–164)	131 (94–186)	−0.116	127 (79–-178)	122 (78–183)	−0.006
Dose of phentolamine, mg	4.0 (0.0–14.5)	2.0 (0.0–12.0)	0.020	3.0 (0.0–13.0)	2.0 (0.0–11.0)	0.130
Dose of esmolol, mg	80.0 (0.0–200.0)	50 (0.0–130.0)	0.117	50.0 (0.0–180.0)	60 (0.0–143.0)	−0.085
Estimated blood loss, ml	100 (50–500)	100 (50–500)	−0.034	100 (50–500)	100 (50–450)	−0.039

Data are the mean ± standard deviation, number of patients (percentage), or median (interquartile range)

SD in bold indicates a significant difference between the two groups

*PPGLs* pheochromocytomas and paragangliomas, *SD* standardized difference, *SBP* systolic blood pressure

^a^ An absolute SD of ≥0.233 was considered ‘unbalanced’ [19]

^b^ Continuous or episodic hypertension with at least one of ‘triad’ symptoms (headaches, palpitations, sweating) at the first clinic visit

^c^ Including calcium-channel blockers, angiotensin-converting enzyme inhibitors, and/or angiotensin II-receptor blockers

^d^ Included retroperitoneal and transperitoneal laparoscopic approaches

### Perioperative hemodynamics and outcome

**Fig. 2:**
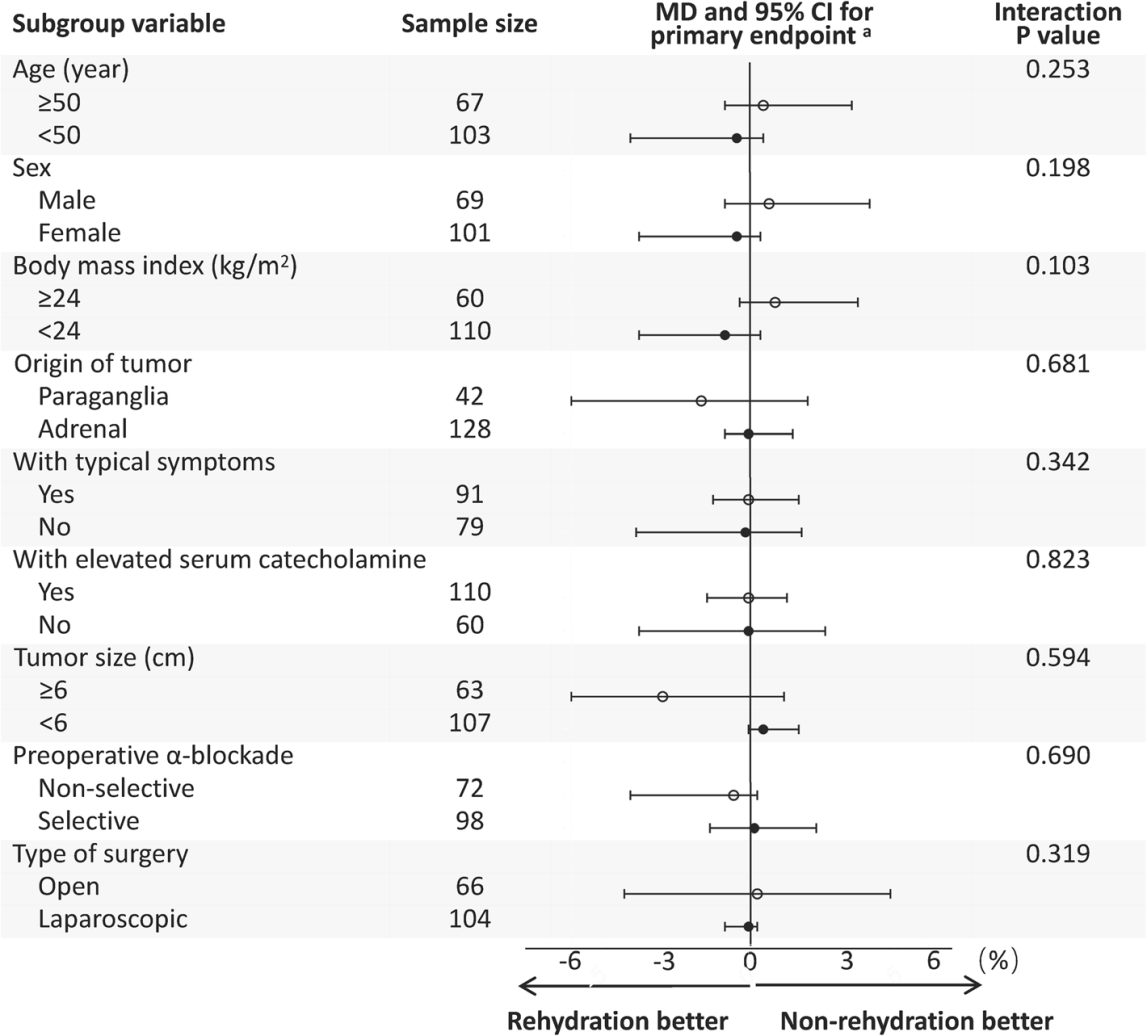
Modified effects of perioperative variables on the association between preoperative intravenous rehydration and the primary endpoint ^a^ in the propensity score-matched cohort. CI, confidence interval; MD, median difference. ^a^ The primary endpoint was intraoperative hypotension, described as the cumulative time of mean arterial pressure < 65 mmHg averaged by surgery duration

The primary endpoint, that is, the cumulative time of mean arterial pressure < 65 mmHg averaged by surgery duration was 3.0% (IQR 0.2–12.2) in patients who had preoperative rehydration compared with 3.8% (0.0–14.2) in patients who did not have preoperative rehydration (median difference 0.0, 95%CI − 1.2 to 0.8, *p* = 0.909) (Table 2). Significant modified effects were not observed between preoperative intravenous rehydration and subgroups, thereby suggesting that the effects of preoperative fluid infusion on different subgroups were similar (Fig. 2).

**Table 2:** Endpoints of the study in propensity score matched patients

Variable	Rehydrated group (***n*** = 85)	Non-rehydrated group (***n*** = 85)	Median difference (95%CI)	***P*** Value
**Primary endpoint**				
Intraoperative hypotension ^a^, %	3.0 (0.2 to 12.2)	3.8 (0.0 to 14.2)	0.0 (−1.2 to 0.8)	0.909
**Secondary endpoints**				
Other perioperative hemodynamic parameters				
Total equivalent dose of catecholamine ^b^, mg	2.7 (0.0–71.7)	0.0 (0.0–38.5)	0.0 (0.0 to 0.0)	0.563
Volume of intraoperative fluids, ml	2800 (2000 to 5300)	3400 (2200 to 4650)	− 250 (−800 to 300)	0.358
Intraoperative tachycardia ^c^, %	3.0 (0.2 to 12.2)	4.3 (0.0 to 14.2)	0.0 (−1.2 to 0.8)	0.641
Intraoperative hypertension ^d^, %	7.9 (1.8–18.4)	7.1 (1.7–16.8)	0.4 (−1.6 to 3.5)	0.629
Postoperative hypotension ^e^	22 (25.9%)	21 (24.7%)		0.860
Postoperative diuretics use	12 (14.1%)	9 (10.6%)		0.484
Early outcome during hospitalization				
ICU admission	58 (68.2%)	56 (65.9%)		0.744
MV in ICU	46 (54.1%)	37 (43.5%)		0.167
Occurrence of complications	13 (15.3%)	7 (8.2%)		0.153
In-hospital death	1 (1.2%)	0 (0.0%)		> 0.999
DOHS, days	14.0 (8.5 to 20.5)	13.0 (8.0 to 20.5)	1.0 (−2.0 to 3.0)	0.563

Data are the median (interquartile range), number of patients (percentage)

*CI* confidence interval, *DOHS* duration of hospital stay, *HR* heart rate, *ICU* intensive care unit, *MV* mechanical ventilation, *SBP* systolic blood pressure

^a^ The cumulative time of mean arterial pressure < 65 mmHg averaged by surgery duration

^b^ Total equivalent dose = (dopamine dose) + (dobutamine dose) + (epinephrine dose × 100) + (norepinephrine dose × 100) [17]

^c^ The cumulative time of HR > 90 beats/min averaged by surgery duration

^d^ The cumulative time of SBP > 160 mmHg averaged by surgery duration

^e^ Hypotension that necessitated continuous vasopressor support to maintain SBP > 90 mmHg after surgery

The intraoperative equivalent dose of catecholamine, the volume of fluids given intraoperatively, cumulative time of heart rate (HR) > 90 bpm/surgery duration, and cumulative time of SBP > 160 mmHg/surgery duration were not significantly different between the two groups. The prevalence of postoperative hypotension was not significantly different between the two groups. Postoperative diuretics use was comparable between the two groups (14.1% vs. 10.6%, *p* = 0.484). There were no significant differences between the two groups with respect to the proportion of patients admitted to the ICU and the proportion of individuals who needed mechanical ventilation. The occurrence of complications was not significantly different between the two groups (Supplemental Table 1). The total DOHS was 14.0 [8.5–20.5] and 13.0 [8.0–20.5] days in patients who had preoperative intravenous rehydration and those who did not, respectively (*p* = 0.563). One rehydrated patient died after surgery due to a large retroperitoneal tumor and massive blood loss (Table 2).

## Discussion

We demonstrated that perioperative hemodynamics and early outcome were not significantly different between patients who had intravenous rehydration and patients who did not have intravenous rehydration before PPGLs resection.

Heterogeneous intravenous rehydration regimes are adopted in different medical centers. The endocrine society guideline recommended continuous administration of 1–2 l of saline starting in the evening before the surgical procedure [2]. However, this recommendation is based on expert consensus rather than clinical research. In clinical practice, we rarely provide an infusion at night to avoid affecting the sleep quality of surgical patients. Most medical centers choose to rehydrate in the daytime instead. Patients did not receive any infusion therapy before surgery in studies by Niederle et al. [15] or Desmonts et al. [21]. Gunawardane et al. [22] and Buitenwerf et al. [23] suggested that 1–2 l of intravenous physiological (0.9%) saline should be used to replete intravascular volume 24 h before the surgical procedure. Wang et al. [24] and Wu et al. [25] recommended rehydrating patients with crystalloids and colloidal fluids for 3–7 days preoperatively. The most commonly used fluid replacement regimen reported in several studies [26–29] was 2000 ml per day for 2 consecutive days before the surgical procedure, which was why we defined preoperative intravenous rehydration as a receipt of ≥2000 ml daily for ≥2 days in the present study.

Three factors may weaken/offset the volume expansion effect elicited by preoperative fluid administration. First, relative intravenous hypovolemia after α-blockade triggers the physiological mechanism of thirst, so blood volume is supplemented by drinking water, which is a voluntary action by the patient without the need of medical advice [30]. Second, crystalloid solutions can cross healthy semipermeable capillary membranes freely; only one-fifth of the infused volume is retained in the vessel. Colloid solutions are more effective in expanding intravascular volume because they are retained within the intravascular space and maintain the colloid oncotic pressure, but the volume expansion effect does not exceed 24 h [31]. Third, anesthesiologists usually implement a mild-overload strategy before anesthesia induction and tumor resection to attenuate post-induction and post-resection hypotension, which possibly weakens the role of preoperative rehydration. In the present study, preoperative rehydration did not provide any improvement in hemodynamics and early outcome, which supported the above arguments.

The necessity for preoperative intravenous rehydration has been doubted. Sjoerdsma and colleagues [32] evaluated plasma volume before and after antihypertensive therapy and after tumor removal using human albumin labeled with ^125^I. Compared with the pre-therapy measurement, the mean plasma volume increased by 11 and 6% following antihypertensive therapy and tumor resection, respectively. Such changes in blood volume were of little clinical relevance and could be restored to normal by physiological regulation of the human body. Lentschener et al. [33] observed no significant difference in mortality prevalence when intravenous fluids were administered guided by arterial blood pressure, which suggested that prophylactic liberal infusion of fluids may not improve outcome in patients undergoing PPGL resection. Mallat and colleagues [34] evaluated the respiratory variation of systolic arterial blood pressure (Δdown) in PPGL patients. They found no significant change in Δdown and no correlation between individual change in systolic arterial pressure or Δdown after tumor resection. Those data suggested that decreased arterial tone (but not reduced preload) was likely a predominant mechanism of hypotension. Niederle et al. [15] implemented goal-directed fluid therapy undertaken by esophageal Doppler ultrasound. They found that vasoplegia, but not hypovolemia, was detected after tumor resection. Iijima et al. [35] revealed that an increased circulating blood volume did not prevent hypotension after pheochromocytoma resection. In view of the evidence stated above, preoperative intravenous rehydration is not likely to be responsible for intraoperative/postoperative hypotension or adverse outcome.

It’s worth noting that indicators of hypervolemia, e.g., presence of pleural effusion and the use of diuretics were not different between the two groups in our study. Three reasons may explain this result. First, most of the PPGLs cases are young patients with normal cardiac and renal function. Excessive fluid can be quickly eliminated by urination, thereby the effectiveness of preoperative fluid expansion may not be sustained in the postoperative period. Second, preoperative fluid overload may not be as detrimental as intraoperative fluid overload. Vascular endothelial integrity plays a crucial role in maintaining vascular volume. In the intraoperative scenario, surgical manipulation, oxidative stress, circulating toxins, inflammatory mediators, and acute hyperglycemia can lead to the breakdown of the endothelial glycocalyx, thus, increasing vascular permeability, resulting in interstitial fluid accumulation and adverse outcome [36]. However, this pathophysiological process is uncommon in the preoperative period. Third, the relatively small sample size may have rejected the statistically significant difference between the two groups.

Due to the availability of effective pharmacological agents, advanced surgical techniques and anesthetic management, the mortality associated with PPGL resection has decreased sharply from 25% in the ‘pioneer period’ [37] to 0.5% nowadays [12]. An ongoing debate surrounding the necessity of preoperative preparation, including preoperative a-blockade and liberal replacement of fluids, springs up constantly [3, 9–11, 15, 33–35, 38–40]. Growing evidence supports the notion that favorable outcome could be achieved through meticulous monitoring of blood pressure, careful surgical dissection and gentle manipulation of tumors, limited intraabdominal pressure, administration of potent, fast-acting antihypertensive drugs, and appropriate fluid management in the absence of preoperative preparation [11, 38, 39]. Intraoperative attention to volume status and expertise in hemodynamic management are likely to be more important than specific preoperative rehydration [3]. Today, with the emergence of various hemodynamic monitors, abandoning the empirical strategy of liberal fluid administration to prevent hypotension and adopting a goal-directed fluid therapy can reduce volume overload effectively [15, 41]. A timely reappraisal of our current practices is necessary.

Our study had several main limitations. First, this was a retrospective study. Even though PSM was adopted to minimize the risk of a bias and increase the reliability of conclusions, confounding factors and a selective bias might exist. Second, studies [5–7] have pointed out that preoperative intravenous rehydration might alleviate postural hypotension after α-blockade. Unfortunately, data of postural hypotension were not documented in the EMRs of our hospital. Third, although the year of surgery was well-matched in the present study, a long period of recruitment may have revealed the diversity of clinical practice. Fourth, only serum catecholamine concentrations, having a lower sensitivity and specificity as compared to metanephrines and urinary catecholamines [42], were routinely tested in our center (in a relevant amount of patients only qualitative). Thus, serum catecholamine level could not be included as metric variable into PSM, potentially causing bias. Fifth, preoperative total amounts of urinary output and oral fluid intake were not documented in the EMRs, preoperative fluid balance could not be calculated or evaluated. Sixth, although pheochromocytomas and paragangliomas share many features including their manifestations, common cell of origin, and catecholamine-producing, they are different in many clinical, biochemical, and genetic aspects [43]. Based on this variety, a generalized recommendation of rehydration for all patients seems to be inappropriate. In Fig. 2, a significant interaction effect was not observed between preoperative intravenous rehydration and the origin of tumors (*P* = 0.681), thereby suggesting that the effect of preoperative fluid infusion on pheochromocytoma and paraganglioma was similar. However, the interaction results in the present study are only hypothesis−generating because confounding has not been controlled for within the subgroups. Seventh, post-hospital-discharge follow-up was not conducted, so the efficacy of preoperative rehydration upon long-term outcome could not be evaluated.

## Conclusions

This was the first study to assess the usefulness of preoperative rehydration upon perioperative hemodynamics and early outcome by comparing rehydrated patients and non-rehydrated patients directly. For patients with PPGLs, preoperative intravenous rehydration failed to optimize perioperative hemodynamics or improve early outcome. Our study suggests it is time to reconsider the necessity of preoperative fluid replacement for patients diagnosed with PPGLs. A prospective randomized controlled study is warranted to confirm our findings.

## Supplementary Information

**Additional file 1: Supplemental Table 1.** Complications during hospitalization in propensity score matched patients.

## Data Availability

The datasets generated and/or analyzed during the current study are not publicly available due to the manuscript has not been received yet but are available from the corresponding author on reasonable request.

## Abbreviations

CI: Confidence interval
DOHS: Duration of hospital stay
EMRs: Electronic medical records
EQUATOR: Enhancing the QUAlity and Transparency Of health Research
HR: Heart rate
ICU: Intensive care unit
IHD: Intraoperative hemodynamic instability
IQR: Interquartile range
MV: Mechanical ventilation
PPGLs: Pheochromocytomas and paragangliomas
PSM: Propensity score matching
SBP: Systolic blood pressure
SD: Standardized difference
