# Supplementary material for: Preoperative intravenous rehydration for patients with pheochromocytomas and paragangliomas: is it necessary? A propensity score matching analysis
Source: BMC Anesthesiol. 2020 Nov 30;20:294. doi: 10.1186/s12871-020-01212-6 (PMC7702709; doi:10.1186/s12871-020-01212-6)
Supplement: Supplementary file 1 — Additional file 1: Supplemental Table 1. Complications during hospitalization in propensity score matched patients. [file 12871_2020_1212_MOESM1_ESM.docx]

**Supplemental Table 1. Complications during hospitalization in propensity score matched patients**

| **Variable** | **Rehydrated group**  **(n = 85)** | **Non-rehydrated group**  **(n = 85)** | **P value** |
| --- | --- | --- | --- |
| Stroke ^a^ | 0 (0.0%) | 1 (1.2%) | >0.999 |
| New onset arrhythmia ^b^ | 1 (0.0%) | 1 (1.2%) | >0.999 |
| Pulmonary complication | 5 (5.9%) | 1 (1.2%) | 0.210 |
| Pulmonary infection ^c^ | 1 (1.2%) | 0 (0.0%) | >0.999 |
| Pleural effusion ^d^ | 3 (3.5%) | 1 (1.2%) | 0.621 |
| Aspiration pneumonia ^e^ | 1 (1.2%) | 0 (0.0%) | >0.999 |
| Pulmonary embolism ^f^ | 2 (2.4%) | 0 (1.2%) | 0.497 |
| Acute kidney injury ^g^ | 10 (11.8%) | 4 (4.7%) | 0.161 |
| Urinary-tract infection ^h^ | 1 (1.2%) | 0 (0.0%) | >0.999 |
| Surgical bleeding ^i^ | 0 (0.0%) | 2 (2.4%) | 0.497 |

Data are the number of patients (percentage).

^a^ Persistent new focal neurologic deficit confirmed by neurologic imaging.

^b^ New onset atrial fibrillation or paroxysmal supraventricular tachycardia that necessitated medical treatment.

^c^ Required antibiotic therapy and presence of at least one of the following manifestations: increased or color-changed sputum, new or changed pulmonary infiltrates, fever, leucocyte count >12,000/mm^3^.

^d^ Chest radiograph shows a blunt costophrenic angle in the upright position, displacement of adjacent structures, or unilateral opaque vascular shadows in the supine position.

^e^ Symptoms caused by inhalation of stomach contents and chest radiograph indicates irregular patchy fuzzy shadows scattered in both lungs.

^f^ Confirmed by computed tomography pulmonary angiography.

^g^ Diagnosed according to KDIGO criteria: serum creatinine increase ≥0.3 mg/dl within 48 h or ≥1.5 times the baseline within 7 days or urine output <0.5 ml/kg/h ≥6 h.

^h^ Confirmed by urinalysis and urine culture and necessitated antibiotic therapy.

^i^ Bleeding after surgery that required secondary surgical hemostasis.
